# Evaluation of an AI-Assisted Virtual Fracture Clinic System: Common Referral Deficiencies and Outcomes Following Automated Feedback in a Major Trauma Centre

**DOI:** 10.7759/cureus.116556

**Published:** 2026-09-19

**Authors:** Wern Y Ooi

**Affiliations:** 1 Orthopaedics and Trauma, Charing Cross Hospital, London, GBR

**Keywords:** artificial intelligence, quality improvement, referral feedback, venous thromboembolism, virtual fracture clinic

## Abstract

Introduction: Virtual fracture clinics (VFCs) rely on accurate and complete referral information to facilitate effective clinical review. Deficiencies in documentation or errors in the selection of the referral pathway result in additional clinical work.

This study evaluated an artificial intelligence (AI)-assisted VFC referral feedback system that checks referrals against predefined local VFC criteria and provides automated feedback when required information is missing. We discuss the common referral deficiencies identified and improvements to referrals following automated feedback.

Methods: A retrospective service evaluation was conducted at Imperial College Healthcare NHS Trust between 18 December 2025 and 26 March 2026. Referrals were made using a cloud-based system called FollowUp.Clinic, which incorporates an AI-assisted referral feedback function.

The system assesses referrals against predefined local VFC criteria and generates automated feedback when deficiencies are identified. This allows referrers to amend their submissions before clinical review. No patient-identifiable information was transmitted to OpenAI (San Francisco, CA, USA) through the API.

Results: A total of 1,201 referrals were processed. Of these, 901 (75.0%) met the predefined referral criteria without requiring modification, while 300 (25.0%) were flagged for modification. The most frequent deficiency was specific imaging not being requested, identified in 167 referrals (55.7%), followed by missing venous thromboembolism (VTE) documentation in 70 (23.3%), insufficient clinical information in 65 (21.7%), and an incorrect referral pathway in 18 (6.0%). Categories were not mutually exclusive, as referrals could contain multiple deficiencies. Following one round of automated feedback, 282 of 300 initially flagged referrals (94.0%) were amended and subsequently progressed through the VFC pathway. The remaining 18 (6.0%) required redirection to an alternative pathway.

Discussion: Most referral deficiencies identified by the system were modifiable, with 94.0% successfully corrected following a single round of automated feedback. Specific imaging not being requested represented the most frequent deficiency. Missing required imaging could add extra steps before treatment. Missing VTE documentation was the second most frequent issue. In the absence of documented VTE assessment, the VFC team had to assume that this had not been completed, with potential implications for patient safety.

Conclusion: The AI-assisted referral system identified common, potentially modifiable deficiencies in VFC referrals, with 94.0% of initially flagged referrals successfully corrected following a single feedback cycle.

## Introduction

Virtual fracture clinics (VFCs) have become an established model for delivering efficient and streamlined orthopaedic care within the UK National Health Service (NHS). VFC pathways aim to facilitate timely specialist review, reduce unnecessary face-to-face appointments [1-3], and optimise patient flow through trauma services. Effective VFC review relies on accurate and complete referral information, appropriate investigation, and selection of the correct referral pathway.

Deficiencies at the point of referral may have consequences beyond referral quality alone. Incomplete clinical information may require further clarification, while failure to request investigations required by local VFC pathways may introduce additional steps before definitive management can proceed. Similarly, incomplete documentation of clinically important assessments, such as venous thromboembolism (VTE) risk assessment in patients with lower-limb immobilisation, may require further clinical review and patient contact [4]. Identifying these deficiencies while the patient remains under the care of the referring team may therefore provide an opportunity for them to be addressed earlier in the pathway.

Artificial intelligence (AI) has increasingly been explored within healthcare to support clinical workflows and the processing of clinical information [5,6]. Within VFC pathways, AI-assisted systems may provide a mechanism for assessing referrals against predefined criteria and providing automated feedback to referrers when deficiencies are identified. This may allow correctable omissions to be addressed before VFC clinical review while maintaining clinical decision-making within the VFC team. However, evidence evaluating AI-assisted feedback specifically within VFC referral pathways remains limited [7].

This study evaluated an AI-assisted VFC referral system designed to identify potentially correctable referral deficiencies. The primary aim was to characterise the common deficiencies identified within VFC referrals and evaluate outcomes following automated feedback to referrers.

## Materials and methods

Study design and setting

A retrospective service evaluation was conducted at Imperial College Healthcare NHS Trust between 18 December 2025 and 26 March 2026. 

Study population

All VFC referrals received during the study period were included in the service evaluation. All referrals were processed through the AI-assisted referral feedback system; therefore, there were no referrals excluded on the basis of not undergoing system assessment.

Data collection and AI-assisted referral system

Data were extracted from the FollowUp.Clinic electronic referral platform [8]. Variables collected included referral outcomes, reasons for referrals being flagged for modification, and categorisation of deficiencies identified within each referral.

FollowUp.Clinic is a cloud-based electronic referral platform incorporating an AI-assisted referral quality assurance and feedback function. The platform operates in accordance with the NHS Data Security and Protection Toolkit (DSPT). The AI-assisted functionality uses OpenAI (San Francisco, CA, USA) services via an application programming interface (API). No patient-identifiable information was transmitted to OpenAI through the API.

Referral information was assessed against a predefined set of criteria derived from local VFC protocols. These criteria included completeness of clinical information, VTE documentation where applicable, referral pathway suitability, and whether specific imaging required according to the relevant local VFC criteria had been requested.

The system incorporated criteria according to injury type and clinical presentation. These included, for example, checking whether specific imaging had been requested for selected knee, shoulder, proximal humeral, elbow, and calf injuries; whether VTE assessment had been documented where applicable; whether sufficient clinical information had been provided; and whether the referral had been directed to the appropriate clinical pathway. The detailed criteria applied by the system are provided in Appendix 1.

Where a predefined requirement had not been met, automated feedback was generated and returned to the referrer, allowing the identified deficiency to be addressed before clinical review. Where specific imaging was required but had not been requested, the system prompted the referrer to initiate the relevant investigation. This was particularly relevant to the local service model because referring Emergency Department clinicians were able to request these investigations, whereas the nurse-led VFC service was unable to independently initiate the required imaging requests.

The AI-assisted system functioned as a pre-review quality assurance and feedback mechanism. It did not replace clinical review or make definitive clinical management decisions, and referrals progressing through the VFC pathway remained subject to clinical review by the VFC team.

For the purposes of this study, referrals identified by the system as not meeting one or more predefined requirements were classified as being flagged for modification. This represented a request for the referrer to address an identified deficiency and did not represent autonomous clinical rejection from VFC review.

Reasons for referrals being flagged for modification were grouped into four predefined categories: (1) Specific imaging not requested; (2) Insufficient clinical information; (3) Missing VTE documentation; (4) Incorrect referral pathway.

Referrals with more than one identified deficiency were recorded within each applicable category and analysed accordingly.

Outcome measures

The primary outcome was the most frequently identified deficiency among referrals flagged for modification by the AI-assisted system. Secondary outcomes included the frequency of each deficiency category, the proportion of referrals progressing without modification compared with those initially requiring modification, patterns in specific imaging not requested, and the proportion of initially flagged referrals successfully amended following automated feedback.

Data analysis

Descriptive statistical analysis was performed. Categorical variables were summarised using frequencies and percentages. Identified referral deficiencies were analysed both individually and in combination to determine the predominant contributing factors. No inferential statistical analyses were performed.

Ethical considerations

This project was registered as a service evaluation within the NHS Trust. All data were anonymised prior to analysis, and no patient-identifiable information was collected. In accordance with local governance policy, formal Research Ethics Committee approval was not required.

Declaration of competing interests

The authors declare that they have no known competing financial interests or personal relationships that could have appeared to influence the work reported in this paper.

## Results

Overview of referrals

A total of 1,201 VFC referrals were processed by the AI-assisted system during the study period. Of these, 901 referrals (75.0%) met the predefined referral criteria without requiring modification, while 300 referrals (25.0%) were initially flagged for modification.

Referral deficiencies identified

The most common deficiency was specific imaging not requested, identified in 167 of the 300 referrals initially flagged for modification (55.7%). Missing VTE documentation was identified in 70 referrals (23.3%), insufficient clinical information in 65 referrals (21.7%), and an incorrect referral pathway in 18 referrals (6.0%). Some referrals contained more than one identified deficiency; therefore, deficiency categories were not mutually exclusive. The distribution of referral outcomes and identified deficiencies is summarised in Table 1.

**Table 1:** Referral outcomes and identified deficiencies *Percentages for deficiency categories and outcomes following feedback are calculated using the 300 referrals initially flagged for modification as the denominator. Individual referrals could have more than one identified deficiency; therefore, deficiency categories were not mutually exclusive and percentages may exceed 100%. VTE: venous thromboembolism.

Outcome/deficiency	n	%
Total referrals	1201	100
Met criteria without modification	901	75
Flagged for modification	300	25
Specific imaging not requested	167	55.7*
Missing VTE documentation	70	23.3*
Insufficient clinical information	65	21.7*
Incorrect referral pathway	18	6.0*
Successfully amended after one feedback cycle	282	94.0*

Specific imaging not requested

Specific imaging had not been requested in 167 referrals. MRI was the most frequently identified outstanding imaging request (n=121, 72.5%), followed by CT (n=23, 13.8%) and ultrasound (n=21, 12.6%). X-ray was required in one referral (0.6%), while one referral had been submitted without the required initial imaging (0.6%).

Recurring clinical scenarios were identified. MRI requirements predominantly related to knee injuries, CT requirements were frequently identified for proximal humerus fractures, and ultrasound requirements commonly related to shoulder injuries. These represented investigations required according to the predefined local VFC criteria that had not been requested at the time of referral. The distribution of outstanding imaging requirements is summarised in Table 2.

**Table 2:** Specific imaging not requested

Imaging	n	%
MRI	121	72.5
CT	23	13.8
Ultrasound	21	12.6
X-ray	1	0.6
Required initial imaging absent	1	0.6
Total	167	100

Clinical information, VTE documentation, and referral pathway

Missing VTE documentation was identified in 70 referrals and represented the second most frequently identified deficiency. Insufficient clinical information was identified in 65 referrals and included omission of information required to establish the clinical history or apply the relevant VFC referral criteria, most commonly the date and mechanism of injury. 

A further 18 referrals had been submitted through an incorrect referral pathway and required redirection to an alternative service. These referrals were classified solely as incorrect-pathway referrals and did not overlap with the other deficiency categories.

Outcomes following automated feedback

Of the 300 referrals initially flagged for modification, 282 (94.0%) were amended following a single round of automated feedback and subsequently progressed through the VFC pathway. The remaining 18 referrals (6.0%) had been submitted through an incorrect referral pathway and required redirection to an alternative service.

## Discussion

The principal finding of this study was that a substantial proportion of VFC referrals contained potentially modifiable deficiencies. Of 1,201 referrals, 300 (25.0%) were initially flagged for modification, and 282 of these (94.0%) were successfully amended following a single round of automated feedback and subsequently progressed through the VFC pathway. Specific imaging not requested was the most frequent deficiency, followed by missing VTE documentation and insufficient clinical information. These findings suggest that most deficiencies identified at the point of referral were correctable and that automated feedback may provide an opportunity to address them before VFC clinical review.

Previous work has similarly shown that the quality and structure of VFC referral information can influence pathway efficiency. Sephton et al. reported significant improvements in referral completeness following the introduction of a digital VFC referral system, together with reductions in referral-to-assessment time and assessment-to-surgery time [2]. The present study extends this concept by evaluating automated feedback at the point of referral, demonstrating that most deficiencies identified by the system could be corrected before VFC clinical review.

Imaging-related referral deficiencies

Specific imaging not requested was the most frequently identified deficiency, occurring in 167 referrals (55.7% of those initially flagged for modification). MRI represented the most common outstanding imaging request, followed by CT and ultrasound. Recurring patterns included MRI requirements for selected knee injuries, CT requirements for proximal humerus fractures, and ultrasound requirements for selected shoulder injuries.

The clinical relevance of these findings relates to the structure of the local VFC pathway. Referrals commonly originate from the Emergency Department (ED), where medical staff can request investigations such as MRI, CT, and ultrasound. In contrast, the nurse-led VFC service is unable to independently initiate these imaging requests. If required imaging has not been requested before referral, identification of the outstanding investigation during subsequent VFC review requires additional clinical input from a clinician authorised to request it. This introduces additional steps into the pathway and has the potential to delay investigation and subsequent management.

Automated identification of outstanding imaging requirements at the point of referral provides an opportunity for the referring clinician to initiate the required investigation while the patient remains under their care. This may facilitate earlier progression through the pathway and reduce the need for subsequent communication between the VFC and referring teams. However, time to imaging and time to definitive treatment were not directly measured in this study. It therefore cannot be concluded that automated feedback reduced treatment delays, and this represents an important outcome for future prospective evaluation.

The recurring imaging deficiencies identified may also provide opportunities for targeted education. Greater awareness of local imaging requirements for commonly referred injuries may reduce the frequency with which referrals require modification and improve familiarity with local VFC pathways. Although previous studies have examined imaging interpretation and the use of AI for fracture detection within VFC pathways, evidence specifically evaluating automated identification of outstanding pathway-specific imaging requests remains limited [7].

VTE documentation and patient safety

Missing VTE documentation was the second most frequently identified deficiency, occurring in 70 referrals (23.3% of those initially flagged). This is particularly important in patients with lower-limb injuries requiring immobilisation, for whom VTE risk assessment and thromboprophylaxis may be indicated in accordance with national guidance [4].

Missing VTE documentation indicated that completion of VTE assessment could not be established from the referral and did not necessarily indicate that an assessment had not been performed. This necessitated subsequent contact with the patient and, where required, return to hospital for completion of VTE assessment and collection of thromboprophylaxis. Missing VTE documentation therefore has implications beyond referral completeness, potentially generating additional patient contact and clinical workload. Where appropriate assessment or indicated thromboprophylaxis has not been completed, there are also important patient-safety implications.

In the wider literature, artificial intelligence and machine-learning approaches have also been investigated in relation to VTE, predominantly for prediction of VTE risk. A recent systematic review and meta-analysis of 27 studies involving 596,092 patients reported promising predictive performance for machine-learning models, although most included studies were considered at high risk of bias [9]. The system evaluated in the present study serves a distinctly different function: it does not calculate VTE risk or determine whether thromboprophylaxis is indicated, but instead identifies absence of required VTE documentation and prompts the referring team to address this. This represents a quality-assurance application of AI supporting completion of an established clinical process rather than replacing clinical VTE risk assessment.

Identification of missing VTE documentation at the point of referral provides an opportunity for the referring team to address the omission while the patient remains under their care. Automated feedback could reduce the need for subsequent patient recall and facilitate timely VTE assessment and provision of thromboprophylaxis where indicated. However, this study did not measure patient recalls, time to thromboprophylaxis, or subsequent VTE events. It therefore cannot establish whether automated feedback resulted in improved VTE-related clinical outcomes.

Clinical information and referral pathway

Insufficient clinical information was identified in 65 referrals (21.7% of those initially flagged). VFC review relies on the clinical information provided by the referring team together with the available imaging. Incomplete information may limit the ability of the VFC team to apply the appropriate pathway and may require further clarification before management can proceed.

A smaller proportion of referrals (n=18, 6.0%) had been submitted through an incorrect referral pathway and required redirection to an alternative service. Referral pathway selection has also been identified as a challenge in other VFC services. Menyah et al. evaluated 191 referrals to a Trauma and Orthopaedic service operating a traffic-light VFC pathway and reported that 39% were considered inappropriate, with a proportion of these referrals having been directed to an alternative pathway when VFC referral would have been appropriate [10]. Although the populations, referral pathways, and definitions of inappropriate referral are not directly comparable, both studies demonstrate that incorrect pathway selection can contribute to inefficiency within acute orthopaedic referral systems. Menyah et al. highlighted education and collaboration between Emergency Department, urgent treatment centre, and orthopaedic teams as potential strategies for improving pathway utilisation [10].

Unlike the other deficiencies identified in the present study, incorrect pathway referrals could not be corrected for progression through the orthopaedic VFC pathway. This included hand injuries, which are managed through a separate specialist Hand VFC under the Plastic Surgery service at our institution. Referrals for these injuries were therefore redirected to the Hand VFC rather than progressing through the orthopaedic VFC pathway. Nevertheless, 282 of the 300 initially flagged referrals subsequently progressed following a single feedback cycle, indicating that most identified deficiencies represented correctable omissions rather than referrals that were fundamentally unsuitable for the orthopaedic VFC pathway.

AI-assisted referral feedback

AI applications within orthopaedics have predominantly focused on areas such as imaging and fracture detection [11]. This pattern is also evident within the VFC literature. In a recent systematic review, Sukhbaatar et al. identified 54 studies evaluating AI tools potentially applicable to VFC pathways, of which 52 focused on X-ray imaging and only two evaluated natural language processing or clinical-text analysis [7]. This highlights the comparatively limited evidence for AI applications involving clinical-text processing within VFC pathways.

The system evaluated in the present study represents a different application of AI within the VFC pathway. Rather than interpreting imaging, autonomously triaging referrals, or making definitive clinical management decisions, the system assesses referral information against predefined local criteria and provides automated feedback when requirements have not been met. Subsequent clinical review and management decisions remain with the VFC team. The high proportion of referrals successfully modified following a single round of feedback suggests a potential role for AI-assisted systems in identifying correctable referral deficiencies before clinical review.

The findings can also be considered alongside previous efforts to digitalise VFC pathways. Bakhiet et al. evaluated a fully electronic patient record-based VFC and reported that an average of 94.5% of patients were assessed within 72 hours, demonstrating high compliance with recommended review times [12]. Although their intervention concerned electronic pathway delivery rather than automated feedback to referrers, both studies demonstrate how digital systems can be incorporated into VFC workflows to support pathway processes. The present study extends this concept by evaluating automated feedback designed to identify and facilitate correction of deficiencies in referral information before VFC clinical review.

In addition to correcting individual referrals, repeated automated feedback may have an educational effect by reinforcing local referral requirements among referrers. Whether this results in sustained improvements in referral quality over time was not assessed in the present study. It is also important to recognise that this study did not compare AI-assisted feedback with alternative approaches such as mandatory electronic referral fields, conventional rule-based validation, or direct clinician feedback. The findings therefore demonstrate the outcomes associated with the system as implemented but do not establish that AI is superior to other methods of referral quality assurance.

Clinical and service implications

The findings highlight potentially modifiable aspects of VFC referral quality with implications for both patient care and service efficiency. Importantly, several of the identified deficiencies required actions that were more readily undertaken by the referring team than by the VFC service itself.

The timing of feedback may therefore be particularly important. Identifying deficiencies while the patient remains under the care of the referring team provides an opportunity for clinicians with direct access to the patient and the ability to initiate investigations or treatment to address the problem before the referral progresses to VFC review. This has the potential to reduce duplicated clinical work, avoid unnecessary patient recall, and reduce delays in the pathway.

Limitations

This study has several limitations. It was conducted within a single NHS Trust and reflects local VFC referral criteria and service organisation, which may limit generalisability. The retrospective design also limited assessment to information contained within submitted referrals rather than the complete clinical encounter. The accuracy of system-generated flags was not independently assessed; therefore, false-positive and false-negative rates could not be determined.

Although 94.0% of initially flagged referrals were successfully corrected following automated feedback, downstream clinical and service outcomes were not directly measured. The study therefore cannot determine whether the system reduced time to imaging or treatment, prevented patient recall, improved VTE-related outcomes, or reduced clinical workload. In addition, there was no comparator group; consequently, the findings demonstrate the performance of the system as implemented but do not establish superiority over conventional referral validation or clinician feedback. Prospective comparative studies are required to evaluate these outcomes.

Future research

Future studies should prospectively evaluate whether AI-assisted referral feedback translates into measurable clinical and service benefits. Relevant outcomes include time to required imaging, time to definitive management, rates of patient recall for incomplete VTE assessment, time to thromboprophylaxis, additional clinical contacts, clinician workload, and cost-effectiveness.

Further work should also examine whether repeated automated feedback produces sustained improvements in referral quality over time. A reduction in recurring deficiencies may indicate an educational effect among referrers. Multicentre evaluation would help determine whether similar systems can be adapted to different VFC pathways and service configurations.

## Conclusions

This study demonstrated that an AI-assisted VFC referral system identified common, potentially modifiable deficiencies in referrals, with 282 of 300 (94.0%) initially flagged referrals successfully corrected following a single cycle of automated feedback. Specific imaging not requested was the most frequent deficiency, while missing VTE documentation represented an important patient-safety concern.

By identifying these deficiencies before VFC clinical review, automated feedback allowed referring clinicians to address outstanding requirements before specialist review. Further prospective evaluation is required to determine whether these improvements in referral quality translate into measurable improvements in patient outcomes or service delivery.

## Appendices

Appendix 1: Predefined referral criteria used by the AI-assisted system

Check there is enough information to work out approximately when the problem began. If the injury is to the hand, the patient should instead be referred to the specific hand virtual clinic. If there is some immobilisation applied to a leg and the patient is over 16, check that there has been a thromboembolism risk assessment or that an anticoagulant has been supplied. There is no need for referrers to include imaging reports. For knee injuries, check that the referrer has requested outpatient MRI if there is suspicion of: patella dislocation, meniscus injury, ligament injury, locking. For shoulder dislocation, check that the referrer has requested outpatient MRI if the patient is aged under 30 and ultrasound if they are 30 to 60 to assess their rotator cuff. For shoulder proximal humerus fractures, check the referrer has organised a CT if the fracture is displaced and in at least three parts, unless the patient has dementia. For elbow dislocations with a fracture, check the referrer has organised a CT. For calf muscle tears, check that the referrer has requested an outpatient ultrasound.
